# Smartphone and Tablet Software Apps to Collect Data in Sport and Exercise Settings: Cross-sectional International Survey

**DOI:** 10.2196/21763

**Published:** 2021-05-13

**Authors:** Matthew Peter Shaw, Liam Paul Satchell, Steve Thompson, Ed Thomas Harper, Carlos Balsalobre-Fernández, Daniel James Peart

**Affiliations:** 1 Sports, Physical Activity and Food Western Norway University of Applied Sciences Sogndal Norway; 2 Department of Psychology University of Winchester Winchester United Kingdom; 3 Academy of Sport and Physical Activity Sheffield Hallam University Sheffield United Kingdom; 4 Number One Strength and Performance Deeside United Kingdom; 5 Applied Biomechanics and Sports Technology Research Group Universidad Autónoma de Madrid Madrid Spain; 6 Department of Sport, Exercise and Rehabilitation Northumbria University Newcastle United Kingdom

**Keywords:** mobile apps, sports, smartphone, mobile phone, questionnaire, survey

## Abstract

**Background:**

Advances in smartphone technology have facilitated an increase in the number of commercially available smartphone and tablet apps that enable the collection of physiological and biomechanical variables typically monitored in sport and exercise settings. Currently, it is not fully understood whether individuals collect data using mobile devices and tablets, independent of additional hardware, in their practice.

**Objective:**

This study aims to explore the use of smartphone and tablet software apps to collect data by individuals working in various sport and exercise settings, such as sports coaching, strength and conditioning, and personal training.

**Methods:**

A total of 335 practitioners completed an electronic questionnaire that surveyed their current training practices, with a focus on 2 areas: type of data collection and perceptions of reliability and validity regarding app use. An 18-item questionnaire, using a 5-point Likert scale, evaluated the perception of app use.

**Results:**

A total of 204 respondents reported using apps to directly collect data, with most of them (196/335, 58.5%) collecting biomechanical data, and 41.2% (138/335) respondents reported using at least one evidence-based app. A binomial general linear model determined that evidence accessibility (β=.35, 95% CI 0.04-0.67; *P*=.03) was significantly related to evidence-based app use. Age (β=−.03, 95% CI −0.06 to 0.00; *P*=.03) had a significant negative effect on evidence-based app use.

**Conclusions:**

This study demonstrates that practitioners show a greater preference for using smartphones and tablet devices to collect biomechanical data such as sprint velocity and jump performance variables. When it is easier to access information on the quality of apps, practitioners are more likely to use evidence-based apps. App developers should seek independent research to validate their apps. In addition, app developers should seek to provide clear signposting to the scientific support of their software in alternative ways.

## Introduction

Advances in smartphone technology have facilitated an increase in the number of commercially available smartphone and tablet apps, enabling the collection of various physiological and biomechanical variables without additional hardware. Smartphones and tablets typically contain a microphone, camera, light sensor, accelerometer, gyroscope, inclinometer, and magnetometer. These hardware components are used in combination with software apps to provide a variety of measurements. Several studies have demonstrated that smartphone and tablet cameras can validly and reliably measure biomechanical variables such as sprint time [1], movement velocity [2,3], and jump height [4]. Both the accelerometer [5] and magnetometer [6] have been used to validly and reliably measure the range of motion in multiple joints, and the inclinometer has been shown to validly and reliably determine break-point angle in the Nordic hamstring exercise [7]. In relation to light sensor hardware, Coppetti et al [8] have examined the ability of smartphones and tablets to measure heart rate via photoplethysmography.

Using smartphones and tablets, sport scientists and coaches are now able to collect data in practical settings, such as during match play or training, more economically. Compared with specialized hardware, commercially available smartphone apps are available at much lower costs or completely free of charge. For example, Romero-Franco et al [1] demonstrated that a smartphone app had comparable reliability and validity to timing gates, costing approximately 400 times more. Mobile technology has the potential to address problems with portability, cost, and time that are historically associated with laboratory-based equipment. However, using smartphones and tablets to measure physiological variables should be performed with caution. For example, there is some inconsistency in the use of software apps to measure heart rate [8], particularly during exercise of various intensities [9]. Furthermore, a recent review by Peart et al [10] highlighted inconsistencies in validity and reliability when estimating body fat percentage using a range of commercially available software [11-13].

Currently, there is very limited existing research that has investigated the use of smartphone or tablet software apps to collect data in sport and exercise settings. Most recently, Bromilow et al [14] surveyed exercise professionals in Australia to examine smartphone use in practice, concluding that smartphone use is highly prevalent in sport and exercise settings, but this is typically for tracking variables. *Tracking* is a term consistent in the sport and exercise literature [15], which refers to apps and software available to log training information. This can include, for example, running distance, resistance exercise repetitions, and heart rate. Typically, users enter this information themselves. Extending on the recent work of Bromilow et al [14], this investigation is the first study that primarily focuses on how practitioners use, or do not use, software apps that use mobile device hardware to collect data directly from the primary source. Furthermore, the existing literature demonstrates inconsistencies in validity and reliability, depending on the type of variable collected, and practitioners should, therefore, be critical in their selection of apps used to collect data. We currently do not fully understand whether practitioners collect data using mobile devices or if they do so using valid and reliable apps. Therefore, the primary aim of this investigation is to explore practitioners’ use of smartphone or tablet software apps for collecting data. A secondary aim is to examine if practitioners select valid and reliable software apps and what may influence this selection.

## Methods

### Overview

An exploratory descriptive study was conducted to examine the current use of smartphones and tablets in sport and exercise settings, providing detailed information on practitioners’ use of this technology. The study used a multiple-choice questionnaire survey that generated exploratory descriptive statistics of app use. The open survey was electronic, with links to the survey distributed using social media platforms such as Twitter and Facebook. The use of Facebook included distributing the survey in specialist groups such as the various National Strength and Conditioning Association special interest groups. Each coauthor sent emails with the links to the survey within their respective professional networks. A final strategy included advertising the survey during international conference presentations. The study procedure was approved by the institutional ethics committee of Sheffield Hallam University (ER8496574) in accordance with the seventh revision of the Declaration of Helsinki in advance of data collection. Survey responses were stored in password-protected files, where only the investigation team could access them.

### Participants

Participants were required to be older than 18 years and engaged in the sport and exercise industry in either an employed or voluntary role to meet our inclusion criteria. Roles included sport scientist, strength and conditioning coach, physical education teacher, sports coach, and personal trainer. Before completing the questionnaire, participants were directed to the participant information section and informed of their right to withdraw from the study. Participation was voluntary, and no incentives were provided. Respondents were required to provide complete responses to the questions. If a question was not answered, they were not able to move to the subsequent question. Valid consent was obtained if the questionnaire was completed [16].

### Survey Instrument

The survey consisted of a series of multiple-choice questions. The survey was developed using Google Forms, allowing participants to complete it remotely, and responses were automatically captured. The survey was open to any visitor to the survey URL. The questions differed based on the previous responses given. The survey gathered 3 areas of information:

- Demographic information: age, gender, and country of residence
- Industrial experience: area of study, area of employment, years of experience, vocational training, professional accreditation, and populations worked with
- App use: type of data collection (eg, cardiovascular), hardware used, and perceptions of reliability and validity.

Participants were given the opportunity to list all the apps they were currently using in practice. Once the survey was closed for responses, we reviewed all reported apps to identify those that had existing literature evidencing reliability and validity. To do so, a series of searches were conducted in Google Scholar and PubMed using the name of each reported app. Only the name of the app was included to determine if it was featured in any existing literature. Once an app was found in any experimental study, we assessed whether the authors reported an app to demonstrate evidence of both validity and reliability. In this investigation, the term *evidence-based app* refers to any app a respondent reported to use that had peer-reviewed evidence of acceptable reliability and validity.

Participants were required to complete an 18-item questionnaire (Textbox 1) to evaluate their perception of app use. The 18 items were formed following consultation with a panel of sport scientists who were independent of the authors’ team and had expertise in survey design. A draft survey structure went through 2 rounds of feedback, following panel feedback and agreement between the coauthors. All items used a 5-point Likert scale (strongly disagree, disagree, neither agree nor disagree, agree, or strongly agree). These 18-items were generated for the target themes of reliability, validity, cost, and ease of use.

**Smartphone or tablet app perception questionnaire.**

Q1. The apps I use in practice are difficult to use

Q2. Reliability of the apps is important

Q3. It is difficult to determine the validity of the apps I use

Q4. The price is important for me when selecting an app

Q5. I do not deem reliability of apps to be important

Q6. Other equipment is harder to use than apps for collecting the same data

Q7. It is easy to determine the reliability of the apps I use

Q8. I am more likely to use an app I have to pay for

Q9. It is easy to determine the validity of the apps I use

Q10. I do not consider the validity of apps to be important

Q11. The apps I use in practice are easy to use

Q12. It is important that there are reliability studies available for the apps I use

Q13. I am more likely to use an app if it is free

Q14. The price is not important for me when selecting an app

Q15. It is important that there are validity studies available for the apps I use

Q16. It is difficult to determine the reliability of the apps I use

Q17. It is important that apps have validity

Q18. Other equipment is easier to use than apps for collecting the same data

### Statistical Analysis

As many of the survey items could be measuring the same core construct, the patterns of responses to the questionnaire were examined using exploratory factor analysis (EFA). Due to missing responses to the questionnaire, 261 cases were used for the EFA. The R statistical package *jmv* was used [17] to conduct an oblique (*oblimin*) minimum residual method EFA. Parallel analysis of the ascending number of factor models suggested that a 4-factor model fit was most effective, explaining a cumulative 36.91% of the variance, with adequate fit indices (Root mean square error of approximation=0.07; Tucker-Lewis index=0.86; *χ*^2^_87_=195.0; *P*<.001). Factor membership of items was assigned based on the strongest loading of an item onto a factor, with all loadings being at least stronger than 0.30.

The 4 factors that emerged in the data were evidence availability, evidence accessibility, nonevidence use, and resources. Table 1 shows the factor loadings with the strongest loading factor highlighted in italics. It is worth noting that the question, “I am more likely to use an app I have to pay for,” poorly loaded on all factors and was dropped from the analysis. The factor *evidence availability* describes the responses of those who think it is important that evidence of an app’s reliability and validity is available and are focused on the validity of their apps. This is distinct from *evidence accessibility*, which reflects the extent to which respondents could determine the reliability and validity of the apps they use. Further factors detailed *nonevidence use,* where participants did not consider validation or reliability important, and *resource*, which reflected responses indicating that price was more important; free apps were less likely to be used. We retained the participants’ derived factor scores for each factor for analysis. These computed variables were all within the normal range of skewness, despite evidence availability (mean 4.16, SD 0.93; skew=−0.93) having a high average and nonevidenced use (mean 1.71, SD 0.66; skew=0.99) having a low average. Evidence accessibility (mean 3.39, SD 0.83; skew=−0.23) and resource (mean 2.44, SD 0.64; skew=0.17) were also within acceptable response ranges.

**Table 1:** The factor loadings of the exploratory factor analysis on the questionnaire items.

Questionnaire item	Evidence availability	Evidence accessibility	Nonevidenced use	Resource
It is important that there are reliability studies available for the apps I use	*0.91^a^*	−0.04	0.00	0.01
It is important that there are validity studies available for the apps I use	*0.90*	0.02	0.00	0.04
It is important that apps have validity	*0.80*	0.04	−0.06	−0.06
It is easy to determine the validity of the apps I use	0.09	*0.82*	0.10	0.03
It is easy to determine the reliability of the apps I use	0.08	*0.77*	0.20	0.01
It is difficult to determine the validity of the apps I use	0.12	−*0.71*	0.18	−0.03
It is difficult to determine the reliability of the apps I use	0.14	−*0.61*	0.33	−0.01
The apps I use in practice are easy to use	0.07	*0.38*	−0.16	−0.15
I do not deem the reliability of apps to be important	−0.07	0.11	*0.65*	−0.12
I do not consider the validity of apps to be important	−0.30	−0.02	*0.47*	0.14
The apps I use in practice are difficult to use	0.00	−0.27	*0.43*	0.23
Reliability of the apps is important	0.25	0.00	−*0.38*	0.13
The price is not important for me when selecting an app	0.01	0.14	−0.03	*0.56*
The price is important for me when selecting an app	−0.04	0.01	0.17	−*0.49*
I am more likely to use an app if it is free	−0.01	−0.15	0.21	−*0.44*
Other equipment is easier to use than apps for collecting the same data	−0.03	−0.12	0.35	*0.44*
Other equipment is harder to use than apps for collecting the same data	0.09	0.13	0.03	−*0.36*
I am more likely to use an app I have to pay for	0.26	0.14	0.22	0.27

^a^The text in italics highlights the strongest load of each item onto the factors.

Tests of the relationship between the categorical variables in this study (job role, level of education, sports type, level of athletes, coded data type, and perceived data type) were analyzed with chi-square tests of independence (using base R), with additional insight provided by the effect size Cramer *V* (using the R package *questionr*) [18]. Binomial linear models (using base R) were used to test the effect of scale variables (such as responses to the questionnaire) on binary outcomes (such as engagement with evidence-based apps or not).

Where the aforementioned categorical variables were used to test for a difference in conceptually and statistically similar dependent variables (the subscales of the questionnaire), multivariate analysis of variance (MANOVA) was used. The overall multivariate effects were tested with Pillai trace to be robust against violations of assumptions. MANOVA tests were conducted with base R, with the additional inference drawn from 95% CI of the omnibus test effect size, ω^2^, using the *MOTE* package [19]. Where needed, post hoc follow-up tests on the MANOVA would involve analysis of variance and two-tailed Welch *t* tests for pairwise comparisons.

## Results

### Demographic Information

The survey received 335 responses. The mean age of the respondents was 32.9 (SD 9.9) years, with a range of 51 years. Respondents of 31 different nationalities completed the survey, with most of the survey responses received from the United Kingdom (107/335, 31.9%), Spain (107/335, 31.9%), and the United States (44/335, 13.1%). A total of 49 different sports were reported by the respondents. Table 2 provides an overview of respondents’ demographic information.

**Table 2:** Respondents’ demographic information (N=335).

Demographic		Respondent, n (%)
**Sex**		
	Male	277 (82.7)
	Female	55 (16.4)
	Did not disclose	3 (0.9)
**Level of education**		
	High school	3 (0.9)
	Further education	18 (5.4)
	Bachelors	89 (26.6)
	Masters	184 (54.9)
	Doctorate	41 (12.2)
**Area of education**		
	Sport and exercise science	132 (39.4)
	Strength and conditioning	65 (19.4)
	Physical education	50 (14.9)
	Sports coaching	23 (6.9)
	Physiotherapy	13 (3.9)
	Physical activity and health	11 (3.3)
	Sports therapy	8 (2.4)
	Nutrition	5 (1.5)
	Psychology	3 (0.9)
	Other	25 (7.5)
**Current role**		
	Education	76 (22.7)
	Applied sport science	148 (44.2)
	Coaching	89 (26.6)
	Other	22 (6.6)
**Type of sports working with**		
	Team sports	116 (34.6)
	Individual sports	84 (25.1)
	Combination	93 (27.8)
**Level of athletes working with**		
	Professional sport	124 (37.0)
	Amateur sport	89 (26.6)
	Combination	92 (27.5)

### Smartphone and Tablet Use

Information on the general use of smartphones and tablets in sports practice is presented in Table 3. Respondents who answered *yes* to using smartphones and tablet devices were then asked to list apps used in their practice (“What apps do you currently use in your practice—please ensure you only refer to apps that do NOT require additional hardware.”). Many respondents (Table 2) reported using either apps that required connection to additional external hardware (eg, GymAware) or apps with a primary function of logging training and activity data (eg, TeamBuildr). Of the 205 respondents who reported using direct data collection apps, the most frequent response (80/205, 39%) to the most important reason for using apps in practice was ease of use. Of the 205 respondents, 116 (56.5%) reported that the cost of apps was the least important reason for using apps. Among the 75 respondents who reported not using smartphones and tablet apps in their sports practice, the most frequently cited reason for not using them was a preference for other equipment (116/335, 34.6%).

**Table 3:** Smartphone and tablet use (N=335).

Characteristics			Respondent, n (%)
**Use smartphones or tablets in sports practice**			
	Yes	260 (77.6)	
	No	75 (22.4)	
**Type of app use in sports practice**			
	Only use apps with direct data collection	150 (44.8)	
	Use a combination of both direct and nondirect data collection apps	55 (16.4)	
	Only use apps with nondirect data collection (tracking apps)	40 (11.9)	
	Only use apps not compatible with a smartphone or tablet (ie, software that functions with PC)	15 (4.5)	
**Type of data collected using apps in sports practice**			
	Only collect anthropometric data	7 (2.1)	
	Only collect physiological and nutritional data	4 (1.2)	
	Only collect biomechanical data	153 (45.7)	
	Collecting a combination of data types	41 (12.2)	

### Types of Data Collection

**Figure 1:**
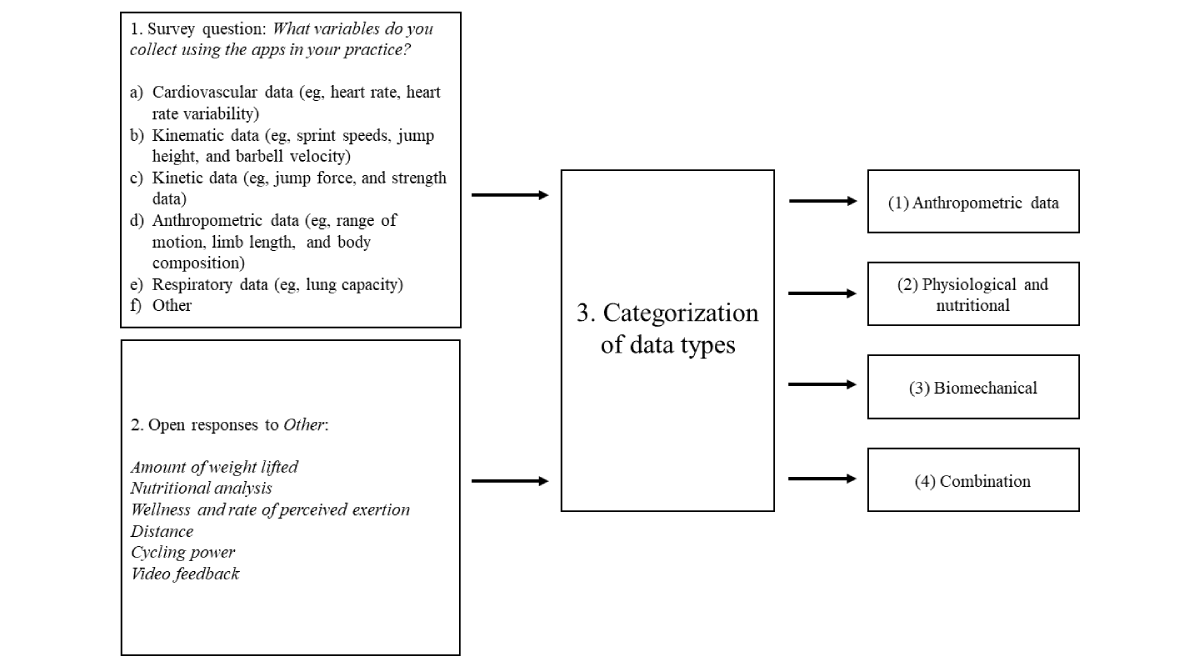
Categorization of data types from survey responses with examples of open-text responses to the "other" option.

Respondents who reported using a smartphone and/or tablet in their sports practice were asked to report what data they collected. We created 4 categories of data use based on these self-reports: (1) anthropometric (eg, joint range of motion, body composition, and limb length), (2) physiological and nutritional (eg, heart rate, heart rate variability, and dietary analysis), (3) biomechanical (eg, kinematic and kinetic data such as sprint speed, jump height, barbell velocity, and force data), and (4) a combination of the abovementioned 3 categories. Data types were coded from the reported apps to investigate how informed respondents were about the meaningful data that could be extracted from the apps (Figure 1). Interestingly, respondents’ perceptions of the data they were collecting were significantly different from the data recorded by the apps (*χ*^2^_4_=230.9; *P*<.001; *V*=0.63). Respondents who were collecting a combination of data types (eg, anthropometric and biomechanical data) accurately reported collecting combined data (41/42, 98%). However, many who exclusively collected biomechanical data reported collecting a combination of data types (81/153, 52.9%) rather than solely biomechanical data (72/153, 47.1%). Furthermore, although many who were collecting no data reported not to collect data (58/75, 77%), other respondents within this group reported collecting biomechanical data (7/75, 9%) or a combination (10/75, 13%) of variables.

### Evidence-Based App Use

Of the reported apps, 13 featured in studies that show no evidence of reliability and validity, whereas 15 apps appeared in studies that demonstrated reliability and validity. Only 1 app was validated but had no literature-demonstrated reliability, and 1 had reliability evidence but no validity evidence; therefore, these 2 apps were included in the 15 evidence-based apps. A total of 58.8% (197/335) respondents did not use any evidence-based apps. Fewer participants reported using 1 evidence-based app (95/335, 28.4%), 2 evidence-based apps (26/335, 7.7%), 3 evidence-based apps (12/335, 3.5%), and 4 evidence-based apps (2/335, 0.6%), and 3 respondents reported using 5 evidence-based apps; no one reported using more than 5 evidence-based apps. Given the limited variability in the number of apps used, we opted not to use the number of apps used as a variable for analysis. Rather, for more robust statistical analysis, the participants were dichotomized into *uses any evidenced-based apps or not*. This effectively presents the greatest behavioral distinction in our sample—engagement with apps or not.

**Figure 2:**
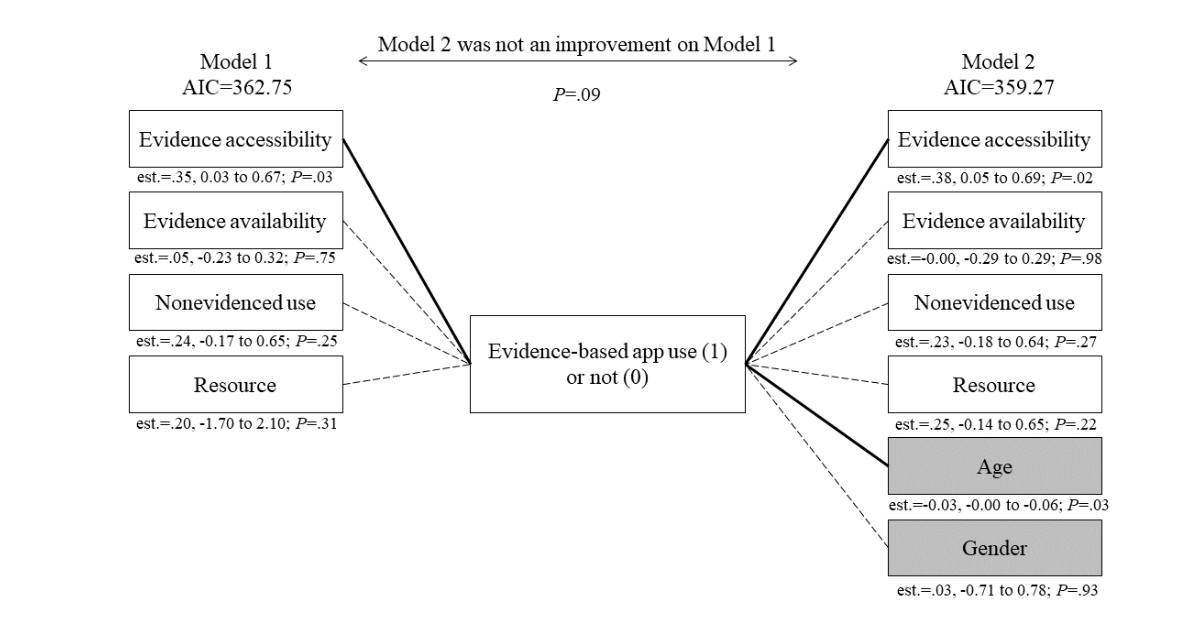
A visual presentation of the results of the 2 linear binomial models. “Est.” is the unstandardized β value predicting evidence-based app use (1) as opposed to not using evidence-based apps (0). Significant predictors are denoted by bold black lines, and nonsignificant predictors are denoted by dotted thin lines. Comparative model fits using the AIC are presented at the top, and the comparison of model fit tests by chi-square tests of variance is explained. AIC: Akaike Information Criteria.

A binomial general linear model using base R (R Foundation for Statistical Computing) was built to test the effect of the questionnaire factors on the use of evidence-based apps. The findings of the first model are summarized in Figure 2. Of the 4 factors (evidence availability, evidence accessibility, nonevidenced use, and resource), the only significant relationship with evidence-based app use was a higher score on evidence accessibility (β=.35, 95% CI 0.04-0.67; *P*=.03), that is, those who reported that it was easier to determine the validity and reliability of the apps were more likely to use those that had an evidence base. Respondents who found it more difficult to evaluate the evidence base of apps were less likely to use the evidence-based apps. None of the other questionnaire factors were significant (Figure 2).

A second model built using the same variables, with the addition of age and gender, was used to examine the general effect of respondent demographics and whether this affected evidence-based app use. This model did not show a significant improvement in explaining the variance in evidence-based app use (Figure 2). The summary of the second model again showed that evidence accessibility has a significant effect. Age had a significant negative effect on evidence-based app use (Figure 2), with younger adults more likely to use evidence-based apps.

Respondents reported information on their job role, education level, and types of athletes they worked with. As these were all discrete nominal variables with no numerical hierarchy—with more than 2 states—they were not included in the linear models. We tested the effect of athlete level (professional, amateur, combined, or no athletes) on engagement with evidence-based apps but found no differences (*χ*^2^_3_=1.3; *P*=.74; *V*=0.06). There were also no differences in the role of the respondent (education, applied sport science, coaching, or other; *χ*^2^_3_=5.2; *P*=.16; *V*=0.01), the type of athlete worked with (team sport, individual, combination, or nonathlete; *χ*^2^_3_=3.8; *P*=.28; *V*=0.01), or the level of education (bachelors, masters, or doctoral degree; *χ*^2^_3_=0.2; *P*=.89; *V*=0.03).

There was variability in engagement with evidence-based apps depending on the type of data being collected (*χ*^2^_2_=145.5; *P*<.001; *V*=0.67). This was explained primarily by the fact that those who were not collecting any data were predominantly using nonevidence-based apps (53/55, 96%) and those who were collecting combined data types preferred evidence-based apps (40/41, 98%) to nonevidence-based apps. Participants solely collecting biomechanical data were split between those using nonevidence-based apps (66/153, 43.1%) and those using evidence-based apps (87/153, 56.9%). In general, those who were focused on collecting more complex data preferred to use apps with a clearer evidence base.

### Questionnaire Responses by Demographics

Given that the questionnaire responses explained variance in engagement with evidence-based apps, it was of further interest to demonstrate any effect of professional activity and training on the questionnaire. There was no general effect of the type of athlete a respondent worked with and their responses to the questionnaire factors (Pillai trace=0.08; *F*_12,765_=1.67; *P*=.07; ω^2^=0.02, 95% CI 0.00-0.03). Similarly, there was no effect of level of athlete on responses to the questionnaire factors (Pillai trace=0.05; *F*_12,765_=1.07; *P*=.39; ω^2^=0.00, 95% CI 0.00-1.00), and there was no effect of respondents’ level of education on responses to the questionnaire factors (Pillai trace=0.04; *F*_4,244_=2.33; *P*=.06; ω^2^=0.02, 95% CI 0.00-0.05).

Interestingly, the respondents’ scores on the questionnaire factors varied by the type of data they were collecting (Pillai trace=0.11; *F*_8,488_=3.51; *P*<.001; ω^2^=0.06, 95% CI 0.01-0.09). This multivariate effect was explained univariate effects of data type on evidence accessibility (*F*_2,246_=4.63; *P*=.01; ω^2^=0.02, 95% CI 0.00-0.07) and evidence availability (*F*_2,246_=7.22; *P*<.001; ω^2^=0.04, 95% CI 0.00-0.09), but there was no effect of data type on nonevidenced use (*F*_2,246_=1.24; *P*=.29; ω^2^=0.00, 95% CI 0.00-0.02) or resource (*F*_2,246_=1.72; *P*=.18; ω^2^=0.00, 95% CI 0.00-0.03).

Participants collecting combined data types were those respondents with higher evidence accessibility scores (mean 3.76, SD 0.77), that is, they were those who indicated that it was easy to determine the evidence basis of the apps. This was demonstrated in subsequent Welch *t* tests, showing that those collecting combined data scored higher on evidence accessibility than those collecting biomechanical data (mean 3.33, SD 0.82; t_66.85_=3.08; *P*=.003; Cohen *d*=0.44, 95% CI 0.19-0.89) or those collecting no data (mean 3.31, SD 0.87; t_91.49_=3.08; *P*=.009; Cohen *d*=0.55, 95% CI 0.14-0.96). Those collecting biomechanical data and those collecting no data did not differ from each other (t_90.59_=0.19; *P*=.85; Cohen *d*=0.03, 95% CI −0.34 to 0.28). The differences in evidence availability followed the same pattern. Respondents who considered reliability and validity more important were those collecting combined data (mean 4.60, SD 0.75). Welch *t* tests demonstrated that those collecting combined data scored higher on evidence availability than those collecting biomechanical data (mean 4.17, SD 0.90; t_73.89_=3.13; *P*=.003; Cohen *d*=0.45, 95% CI 0.20-0.90) or those collecting no data (mean 3.90, SD 0.99; t_93.9_=3.96; *P*<.001; Cohen *d*=0.82, 95% CI 0.39-1.24). Again, respondents collecting biomechanical data and respondents not collecting data did not differ from each other (t_88.18_=1.78; *P*=.08; Cohen *d*=0.25, 95% CI −0.03 to 0.59).

## Discussion

### Principal Findings

This study aims to review the prevalence of mobile app use in the sport and exercise science industry, with a particular focus on apps with an evidence base to support their app for direct data collection. The main findings were that (1) 61.2% (205/335) of respondents reported using apps to directly collect data, (2) there was a misunderstanding in some users regarding the type of data being collected by the app, (3) biomechanical data were the most frequently collected type of data, (4) more than half of those using apps to collect data were doing so with apps that had no evidence base, and (5) perceived evidence availability and evidence accessibility had the strongest effects on evidence-based app use.

Consistent with the findings from the study by Jospe et al [20], the most frequently reported reason for using smartphone apps was a perceived ease of use, which is consistent with the broader literature on mobile technology [21,22]. However, the most frequently cited barrier to not using smartphones and tablets in practice was a preference for other equipment. Furthermore, one-fifth of the respondents reported not using apps because of a lack of compatibility with their current resources. This has previously been highlighted by Ravenek and Alvarez [23], whereby practitioners may not have the appropriate infrastructure to support mobile device use (eg, internet connectivity). Consequently, those who do not use apps in their sports practice may be hindered by structural and operational constraints specific to their respective workplaces rather than a lack of motivation to engage with smartphones. Conversely, using smartphones within health and other related contexts is still considered a new concept [23,24], and some practitioners may be skeptical of new technology, particularly if they perceive a lack of knowledge in using smartphone apps. Evidence of this concern is consistent in the broader literature, as practitioners are uncomfortable in both prescribing apps to clients and patients and using them in personal practice if they feel they do not possess the appropriate prerequisite knowledge [20,24,25]. Another consistent theme in the existing literature is greater smartphone use by younger respondents [26]. In this study, we found that age had a significant negative effect on evidence-based app use, whereby younger adults were more likely to use evidence-based apps. This investigation has predominantly focused on how mobile technologies are used in sport and exercise practices, as opposed to why the technology is used. Further investigations are therefore required to examine the possible reasons for not using smartphone and tablet technology.

To our knowledge, this is the first investigation to explore the prevalence of smartphone and tablet use for direct data collection, that is, no additional hardware in conjunction with app use. Respondents were asked, “What apps do you currently use in your practice—please ensure you only refer to apps that do NOT require additional hardware?”. A total of 55 respondents did not use apps that used a smartphone or tablet as a direct data collection tool, that is, they did not use the internal hardware of a smartphone or tablet.

Typical responses were those reporting the use of an app that stores information entered by a user. Examples of this include logging repetitions, sets, and loads used in resistance training sessions or recording heart rate determined by an external device typically paired to a smartphone via Bluetooth. When these types of users were removed, 61.2% (205/335) of our sample were using a smartphone or tablet to directly collect data. There were further misunderstandings when respondents were asked to report what types of data they collected (eg, anthropometric, physiological, and biomechanical). For example, some respondents reported collecting both biomechanical and anthropometric data but only listed apps designed to measure biomechanical data, such as jump height and sprint speed. The first explanation for this misunderstanding is that the survey was provided to all respondents in British English only, despite having responses from 31 different countries. Of those that erroneously reported the types of data they were collecting from their respective apps, 51% (50/98) were from a country that did not have English as an official language. Questions can be interpreted differently depending on the language it is asked in [27]. It may have been difficult to understand the difference between recording inputted data and directly collecting data with a smartphone app and the difference in data types. As many of these respondents were from English-speaking countries, a second explanation is a general misunderstanding of which apps they use and their capabilities. Bromilow et al [14] found that 56% of their respondents could not identify which smartphone apps they used.

Despite some misunderstanding of the types of data being collected, the number of respondents reporting the use of a smartphone or tablet was much higher than that reported by Bromilow et al [14], who found that only 9% of their sample used a smartphone for direct data collection. Biomechanical data, such as kinetics related to vertical jump performance, were the most frequent (153/335, 45.7%) type of data collected. The results of our survey suggest that this is because of the perceived availability of peer-reviewed literature. Evidence accessibility was the only significant element of the model for evidence-based app use (β=.35, *P*=.03), and respondents were more likely to use apps with an evidence base if they perceived it was easier to find evidence of validity and reliability. This is unsurprising considering the current wealth of literature that has focused on the validation of apps used to collect various biomechanical variables. MyJump2 is a smartphone app that has been shown to validly and reliably estimate vertical jump performance in multiple populations [28,29]. The app has featured so significantly in the peer-reviewed literature that a narrative review has been provided by Sharpe et al [30]. There is significant cost and expertise required for collecting these type of data using more traditional methods such as a force plate [10], which may explain why cost-effective and user-friendly apps—investigations of their respective validity and reliability—are popular in this particular discipline.

In contrast, although there is some evidence demonstrating valid and reliable cardiovascular measures, such as heart rate variability [31], Muntaner-Mas et al [32] suggest that there is a general lack of peer-reviewed literature on apps related to cardiorespiratory fitness. This is in line with our findings that only 7% of respondents reported using apps to collect physiological data variables. Peart et al [10] suggested that there is now a stable body of research on apps that collect biomechanical data. This seems promising; however, of the respondents stating that they only collected biomechanical data, 43.1% (66/153) did not use evidence-based apps. Therefore, although this area has a number of apps supported by the literature, there is also more choice available and an increased risk of selecting nonvalidated apps. For example, smartphone apps that collect kinematic and kinetic data of barbell exercises continue to be developed and made commercially available for validity and reliability studies. It is, therefore, possible that some users download an app based on popularity rather than their quality, with regard to validity and reliability, as a result of *app overload* [24].

In total, 59.0% (121/205) of the respondents did not use evidence-based apps, that is, where peer-reviewed literature has provided evidence of acceptable reliability and validity. The literature assessing the validity and reliability of mobile device hardware used in other contexts is extremely limited, making it difficult to draw direct comparisons with other contexts. Many apps are commercially available to promote behavior change, such as smoking cessation [33], weight loss [34], and suicide prevention [35]. Haskins et al [33] identified 6 smoking cessation apps with some level of scientific support, of which only 2 featured in any top 50 app lists in web-based app stores. The authors concluded that scientifically informed apps were underutilized. Although not directly comparable, our findings are consistent with other app contexts, showing that many users adopt software apps with no scientific support. There is consensus in the broader literature that there are challenges in highlighting the availability of scientifically informed apps to a user base, and, as demonstrated by our findings, the sport and exercise science community is not an exception to this. The use of evidence-based apps was significantly explained by the *evidence accessibility* factor in our model. This factor reflects the extent to which respondents could determine the reliability and validity of the apps they use, independent of the amount of evidence available. Therefore, even if users had access to scientific information, their self-reported ability to understand evidence was the main driver of their choice to use evidence-based apps. In practical terms, this means that although the existence of evidence is important, whether this evidence is effectively communicated to the consumer is of higher importance. Interestingly, the volume of data collected by apps influenced the likelihood of choosing evidence-based apps. Respondents collecting a combination of data types considered reliability and validity more important when selecting data. We speculate that those who were collecting multiple performance variables would have to use multiple apps and were therefore more aware of data collection apps currently available on the market. Such individuals are potentially more familiar with which apps have an existing evidence base in the literature. This is consistent with the existing literature, which indicates that smartphone app proficiency is more closely related to individual interest rather than the level of education [14]. Our results demonstrated that the level of education did not significantly affect the adoption of evidence-based apps.

### Limitations

The limitations of this study must be addressed. First, a limitation of survey research, in general, is the risk that they are more likely to be completed by people who have a preconceived idea about the topic in question, that is, people who use apps may be more likely to be interested in taking part. For example, Bromilow et al [14] found that 99% of their sample reported using smartphone apps in their sport and exercise science practices. In this study, we found that smartphone and tablet use was less prevalent than previously reported [14], with 78% of respondents reporting the use of a smartphone or tablet in their practice. Both this investigation and the previous investigation by Bromilow et al [14] may possess a nonresponse bias [36], which overestimates smartphone and tablet use in sport- and exercise-related practices. In a related context, Jospe et al [20] suggested a nonresponse bias as a limitation of their investigation of sport dieticians’ use of smartphone apps.

Apps that had their reliability and validity findings reported in the academic literature were considered evidence-based apps for the purpose of this investigation. However, it is possible that the software developers have conducted internal validation testing and calibration. As peer review and publication of academic literature can take a substantial amount of time, it is plausible that some mobile software apps are valid and reliable but have not yet been reported and published in the literature. In relation to this, another potential limitation is that some respondents may not have known the difference between validity and reliability. However, we did not find any differences between the reliability and validity questions in our survey. In addition, we did not ask participants how they may have used reviews and user ratings to inform their app selection. This was beyond the scope of this study, and further research is required to qualitatively investigate how users decide to select the apps they use in practice.

This investigation provides insight into the broad use of mobile technologies to directly collect data in sport-related and exercise science–related fields. The results of this study demonstrate that practitioners show a greater preference for using smartphones and tablet devices to collect biomechanical data such as sprint velocity and jump performance variables. This may be because of a greater prevalence of peer-reviewed literature, which has provided evidence of valid and reliable apps, and because practitioners can access this information. When practitioners perceive that it is easier to determine the quality of apps, this leads to increased adoption of evidence-based apps. Therefore, there are 2 key implications for app developers. First, app developers should seek independent research to validate their apps. Second, app developers must consider how they market their products. Using journal articles to select apps is ineffective [14], and app developers should look to provide clear signposting to the scientific support of their software in alternative ways, such as app store descriptions and social media.

## Abbreviations

EFA: exploratory factor analysis
MANOVA: multivariate analysis of variance
